# Meningococcal Carriage in Military Recruits and University Students during the Pre MenB Vaccination Era in Greece (2014-2015)

**DOI:** 10.1371/journal.pone.0167404

**Published:** 2016-12-01

**Authors:** Kyriaki Tryfinopoulou, Konstantinos Kesanopoulos, Athanasia Xirogianni, Nektarios Marmaras, Anastasia Papandreou, Vassiliki Papaevangelou, Maria Tsolia, Aftab Jasir, Georgina Tzanakaki

**Affiliations:** 1 National Meningitis Reference Laboratory (NMRL), Dept of Public Health, National School of Public Health, Athens, Greece; 2 European Public Health Microbiology Training Programme (EUPHEM), European Centre For Disease Prevention and Control, Stockholm, Sweden; 3 Third Department of Paediatrics University General Hospital ATTIKON National Kapodistrian University of Athens, Athens, Greece; 4 Second Dept of Paediatrics “Aglaia Kyriakou” Children’s Hospital, School of Medicine, National Kapodistrian University of Athens, Athens, Greece; University of Cambridge, UNITED KINGDOM

## Abstract

**Purpose:**

The aim of the study was to estimate the meningococcal carriage rate and to identify the genotypic characteristics of the strains isolated from healthy military recruits and university students in order to provide data that might increase our understanding on the epidemiology of meningococcus and obtain information which helps to evaluate the potential effects on control programs such as vaccination.,

**Methods:**

A total of 1420 oropharyngeal single swab samples were collected from military recruits and university students on voluntary basis, aged 18–26 years. New York City Medium was used for culture and the suspected *N*. *meningitidis* colonies were identified by Gram stain, oxidase and rapid carbohydrate utilization tests. Further characterisation was carried out by molecular methods (multiplex PCR, MLST, WGS).

**Results:**

The overall carriage rate was of 12.7%; 15% and 10.4% for recruits and university students respectively. MenB (39.4%) was the most prevalent followed by MenY (12.8%) and MenW (4.4%). Among the initial 76 Non Groupable (NG) isolates, Whole Genome Sequence Analysis (WGS) revealed that 8.3% belonged to MenE, 3.3% to MenX and 1.1% to MenZ, while, 53 strains (29.4%) were finally identified as capsule null. Genetic diversity was found among the MenB isolates, with 41/44 cc and 35 cc predominating.

**Conclusion:**

Meningococcal carriage rate in both groups was lower compared to our previous studies (25% and 18% respectively) with predominance of MenB isolates. These findings, help to further our understanding on the epidemiology of meningococcal disease in Greece. Although the prevalence of carriage seems to have declined compared to our earlier studies, the predominant MenB clonal complexes (including 41/44cc and 35cc) are associated with invasive meningococcal disease.

## Introduction

Invasive Meningococcal disease (IMD) is a life threatening illness with annual incidence varying from 1–1000 per 100 000 in different parts of the world. *Neisseria meningitidis*, is a pathogen of global significance causing sporadic cases and most importantly periodic epidemics with high mortality rates [1]. IMD is almost exclusively caused by encapsulated meningococci expressing one of 12 serologically distinct serogroups such as A, B, and C and to a lesser extent Y and W, belonging to many diverse lineages. The capsule is an important virulence factor protecting the bacterium from opsonophagocytosis in the bacteremic stages [2].

The human naso-pharynx is a known reservoir for meningococcal asymptomatic carriage. Studies performed in Europe have shown that asymptomatic carriage is an age-dependent phenomenon showing low prevalence in the first years of life, with a sharp increase in teenagers and young adults [3] and also associated with factors such as active and passive smoking, concomitant viral or bacterial respiratory infections, low socioeconomic status and crowding conditions. In closed and semi-closed populations, such as military recruits and university students, transmission increases and carriage rates may then reach higher percentages [1, 4, 5, 6].

The relation between carriage rate and invasive disease is not clearly understood. Carriage studies can provide valuable information on epidemiology, pathogenesis, serogroup distribution and possible transmission patterns, information which helps understanding the potential effects of control programs, such as vaccination [7].

An effective meningococcal vaccine could have multiple benefits, as demonstrated by recent studies on the implementation of conjugated MenC vaccine in UK showing 67% reduction (from 0.45% to 0.15%) in the prevalence of serogroup C carriage in adolescents one year after the introduction of this particular vaccine [8]. Furthermore, a reduction of meningococcal disease incidence was also observed in both vaccinated and non-vaccinated groups, as a probable consequence of enhanced herd protection [9,10]. Thus, when introducing new meningococcal vaccines and assessing their effectiveness, it is also essential to consider their effect on the meningococcal carriage dynamics.

In Greece, the monovalent conjugate vaccine for MenC was introduced in January 2001 and included in the national immunization program in 2005 in older children and adolescents, with estimated vaccination coverage from 20.7% (2001) to 51.4% (2005) [11]. Since April 2011, the quadrivalent meningococcal conjugate vaccine (MCV4) was included in the national immunization programme as a booster dose in adolescents 11–16 years old.

The main objectives of this study were (a) to estimate the meningococcal carriage rate in healthy individuals living under semi-closed conditions (military recruits) and university students aged between 18–26 years old, (b) to identify the genogroups and compare those with previous carriage studies in similar populations in Greece, and (c) to obtain baseline data on genogroups and genotypes that may serve for future comparisons and estimations of the possible effect of immunization with the new multicomponent Men B vaccine (4CMenB) which is not yet broadly implemented in Greece on the dynamics of meningococcal carriage.

## Material and Methods

### Study design and population

Two cross-sectional studies were performed to collect pharyngeal swabs from healthy young adults (military recruits and university students). The studies were carried out in January 2014 in a central military camp in Peloponnese (military recruits) and in May 2015 in Athens (university students). For the military recruits, the study population was defined as all new recruits joining the military camp in specific dates (day 1, and 7 of recruitment). As for the university students, sampling was performed during their annual national conference in Athens and came from different universities all over Greece. It is noted that participants from both groups were sampled on voluntary basis.

Permission for the surveys was obtained from the Ministries of Defence and Education. Prior to sample collection, the participants responded ad hoc to a structured self-administrated questionnaire including information on age, region of origin, smoking habits, recent mild infections of the upper respiratory tract and meningococcal vaccination status for both conjugated vaccines (MenC and MCV4). A written informed consent form was then signed, and a same unique identifier was used in both the inoculation plate and the filled questionnaire.

Sample size was calculated on the assumption of meningococcal carriage rate of 25% (military recruits) and 18% (university students NMRL 2002, unpublished data) as described previously [12]., In order to calculate at 95% confidence level for the anticipated frequencies (*p*), with a margin of error ± 3%, the estimation on the sample size was 800 and 630 for the military recruits and university students respectively (OpenEpi, Version 3).

### Specimen collection and bacterial identification

Pharyngeal single swabs were collected from the posterior wall behind the uvula by trained staff. The swabs were directly plated in New York City Medium (OXOID LTD, Basingstoke, Hampshire, England) and incubated at 37^0^ C in the presence of 5% CO_2_. Culture plates were examined at 24 and 48 hours for suspected *N*. *meningitidis* colonies.

Further identification was carried out by the use of Gram’s stain, oxidase test and rapid carbohydrate utilization tests. All isolates were preserved at -70°C in Heart Infusion Broth for further analysis. Genogroups were determined by multiplex PCR targeting specific genes for MenB, MenC, MenA, MenW/Y in all meningococcal isolates as previously described [13]. Further analysis of isolates negative for genogroup-specific PCR assays was carried out by Whole Genome Sequencing (WGS). In brief, genomic DNA was extracted by the use of GenElute™ Bacterial Genomic DNA Kit (Sigma-Aldrich Co LLC) according to the manufacturer’s instructions. The WGS was performed on purified genomic DNA (gDNA) by parallel high-throughput ‘next generation’ sequencing (NGS) technologies by means of illumina Nextera technology (Illumina Inc., San Diego, CA, USA) according to the manufacturer’s protocol.

Clonal Complexes (cc) were defined as the sequence types (ST) which were grouped by their similarity to a central allelic profile including any ST that matches the central genotype at four or more loci. The cc for MenB and negative for genogroup specific PCR isolates were determined by the WGS analysis and the data were investigated with de novo assembly and population annotation to characterize meningococcal genomes as described previously [14].

### Statistical methods

The present study is a descriptive epidemiological study, endpoints were estimates without formal hypothesis testing. For meningococcal carriage rate estimates, the numerator was the total number of carriers and the denominator was the total number of participants. The odds ratios (OR) between exposed and non-exposed were compared for some known factors associated with meningococcal carriage and the respective 95% confidence intervals were calculated by the use of Open Epi, v3 program. In addition, multivariable analysis using a logistic regression model was performed in order to assess independent associations, while adjusting for potential confounders. All statistically significant factors in the univariate analysis were included in the model (SPSS v.20.0). The Z- test for two population proportions was used and a two-tailed p<0.05 was considered as significant.

### Ethical approval

The study was approved by the Committee of Bioethics and Research Ethics of the National School of Public Health.

## Results

A total of 1420 participants were enrolled; 680 military recruits and 740 university students. Sampling in military recruits was carried out in two stages: 336 were sampled during their 1^st^ day in the military camp, while the remaining 344 were examined 7 days after their admission due to recruitment system in Greece.

According to the questionnaires, the median age for military recruits and university students was 20 and 21 years respectively. Regarding their reported place of origin, the subjects were from all over the country. Only a low number (1.5%) of students were living in the university house of residence while the rest were living in their own apartments.

The majority were non smokers (65.1%; 925/1420). Among the 493 smokers, 77% (379/493) were military recruits, while 23% (114/493) were university students. Nineteen percent (264/1403) had experienced a mild upper respiratory tract infection within 2 weeks prior to sampling.

Available data on vaccination status were recorded mainly for the MenC vaccine; 54.7% (372/680) of the military recruits and 72.2% (534/740) of the university students reported to be vaccinated. The highest vaccination rate was recorded in participants from Attica (58.6 and 76% among the recruits and students respectively). In contrast, regarding the vaccination status on MCV4 vaccine, the majority of the participants (100% and 79.9% of the military recruits and university students respectively) responded that their status was not known (˝S1 Table˝).

Among the 1420 pharyngeal samples, 180 *N*. *meningitidis* isolates were recovered, rendering an overall carriage rate of 12.7%; 15% (103/680) in the military recruits and 10.4% in university students (77/740).

Univariate analysis revealed that age (18–21 years), active smoking and living in crowded conditions were all positively associated to meningococcal carriage. In contrast, mild upper respiratory tract infection was not associated with carriage as shown in ˝Table 1˝. In addition, multivariable analysis revealed that smoking (OR = 1.91, p = 0.000), age group 18–21 (OR = 1.81, p = 0.001) and living in crowded conditions (OR = 1.45, p = 0.04) were independently associated with a higher risk of carriage.

**Table 1:** Factors associated with meningococcal carriage in the studied population.

	Carriers(n = 180)	Non carriers(n = 1240)	OR	95% CI	*p-*value
Associated factor	N (%)	N (%)			
Age group (18–21 years)	132 (73.3)	778 (62.7)	1.633	1.151–2.317	0.005
Active smoking	89 (49.4)	404 (32.63)	2.019	1.473–2.768	<0.001
Living in semi closed conditions^a^	60 (33.3)	295 (23.8)	1.602	1.144–2.242	0.007
Prior mild respiratory tract infection	27 (15)	237 (19.3)	0.739	0.479–1.141	0.168

^a^ University students living in a student residence and recruits examined 7 days after their admission in the military camp (n = 355)

As participants originated from all over the country (˝S2 Table˝); (˝S1 Fig˝), the meningococcal carriage rate in relation to their place of origin was analysed. Univariate analysis revealed that there was no statistically significant association between carriage and place of origin (p = 0.615) (˝S3 Table˝).

The 180 meningococcal isolates belonged to several genogroups. According to the genogroup-specific multiplex PCR, 71 (39.4%) were identified as MenB, 23 (12.8%) MenY, 8 (4.4%) MenW and 2 (1.1%) MenC. For the remaining 76 which were negative for genogroup specific PCR, WGS analysis revealed that 8.3% belonged to MenE, 3.3% to MenX and 1.1% to MenZ, while 53 strains (29.4%) lacked the genes required for capsule synthesis and transport and were identified as capsule null strains (cnl) (˝Table 2˝).

**Table 2:** Genogroup distribution among the carrier isolates in both studied groups.

	**Total**		**Recruits**		**University students**		***p*-value**
Genogroup	n	%	n	%	n	%	
**B**	71	39.4	46	44.7	25	32.5	0.20
**C**	2	1.1	2	1.9	0	0	0.21
**W**	8	4.4	3	2.9	5	6.5	0.25
**Y**	23	12.8	9	8.8	14	18.2	**0.06**
**X**	6	3.3	2	1.9	4	5.2	0.23
**Z**	2	1.1	2	1.9	0	0	0.22
**E**	15	8.3	8	7.8	7	9.1	0.75
**cnl**	53	29.4	31	30.1	22	28.6	0.82
**Total**	**180**	**100**	**103**	**100**	**77**	**100**	

No significant differences were observed between the two examined groups with the exception of MenY which was found in higher proportion in university students at marginal significance level (˝Table 2˝).

MenB was the most predominant in all age groups in both populations; in particular, 44.7% (46/103) and 32.5% (25/77) was identified in military and university students respectively, isolated from carriers all over Greece and was the most prevalent among those from Northern Greece accounting for 60% (18/30) of all isolates recovered from carriers from this region.

**Fig 1:**
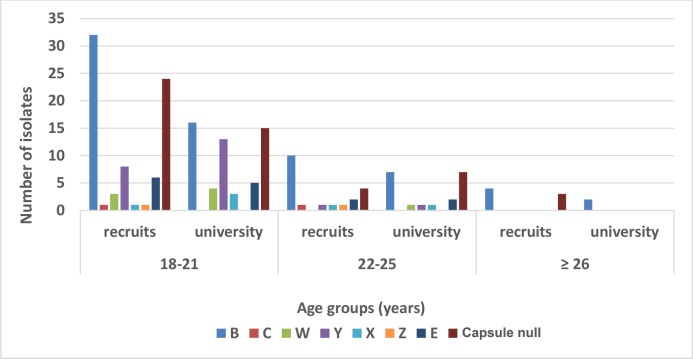
Genogroup distribution by age and examined group

MenY was isolated mostly in the younger age group (18–21 years old), and at a higher proportion in university students compared to military recruits (23.2% vs 10.5%, p<0.05). No significant differences were observed in the distribution of the remaining genogroups in relation to age (“Fig 1”).

**Fig 2:**
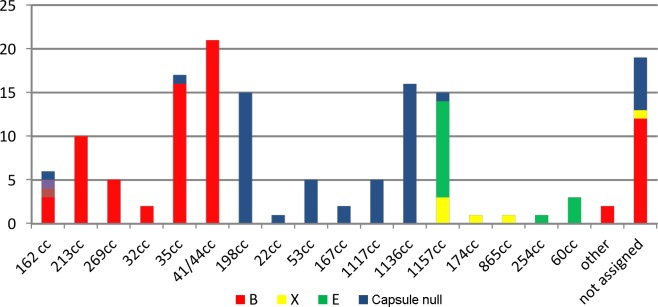
Genogroup assignment of the 145 meningococcal isolates to clonal complexes

Clonal complexes (cc) were determined for 145 isolates belonging either to MenB, or were negative for genogroup specific PCR which were identified as MenX, MenE, MenZ and cnl: 126 were assigned to 19 different cc, while 19 isolates were not assigned to any cc (singletons). Specifically, MenB isolates were distributed into 6 distinct clonal complexes; 41/44cc was the most predominant (21/70; 30%) followed by 35cc (16/70; 22.9%) and 213cc (10/70; 14.3%). Moreover, MenX isolates were distributed into 3 clonal complexes: 1157cc (n = 3), 174cc and 865 cc (1 strain each) and MenE isolates were assigned also to 3 clonal complexes (1157cc being the most prevalent 73.4%; 11/15). Finally, among 53 cnl isolates, 48 were assigned into 9 clonal complexes represented mainly by 1136cc (34%) and 198cc (31.2%) and to lesser extent by 53cc and 1117cc (10.4% each), while 6 isolates remained unassigned (˝Fig 2˝).

## Discussion

In order to study meningococcal pathogenesis, the understanding of *N*. *meningitidis* carriage is fundamental, since it might provide key information in the connection between carriage and disease [8]. Furthermore, extensive typing of invasive and carriage strains is necessary in order to monitor changes in circulating meningococcal strains due to vaccine implementation and possible selection for particular existing strains, since conjugate vaccines against meningococci have been used worldwide with the exception of the recent licensure of serogroup B vaccine [8].

A gradual decrease in incidence has been observed in Greece during the last 20 years. The overall IMD incidence during 1995–1999 was 1.46 per 100 000 and 1.6 per 100 000 during 2000–2005 [15], with a further decline during 2006–2012 (0.71 cases per 100,000 population) and the last three years ranging from 0.61 (2013) and 0.59 (2014) to 0.52 (2015) per 100,000 population, with the disease appearing to be more frequent among children in the 0–4 age group and less frequent in the 5–14 and 15–24 age groups [16].

Several parameters were associated with meningococcal carriage in our study population such as positive association between carriage and younger age, active smoking and living in semi-closed communities which is in accordance with previous studies in UK [4, 17], Norway [18] and Greece [12].

Overall, a lower carriage rate than expected was observed for both groups. Among the military recruits, a lower carriage was observed (15% vs 25%; p<0.05) [12]. This is in line to a similar study from Finland (2004–2005) (2,2% vs 38%) [7]. Furthermore, as shown during 1986–1998 on the European level, prior to vaccine implementation, high carriage rates were observed among the military recruits. For instance, the carriage rates in Denmark were as high as 41–46% during 1992–1993, 38% in Finland (1986), 16–32% in Italy (1988) and 16–24% in Poland (1998) [7].

Similar observations were reported in non-military young adults which, rates ranged from 21.7–36.1% in Czech Republic (1996), 14.6–47% in UK (1997–1998) and 28.3% in Norway (1991) [7].

Moreover, regarding university students, the carriage rate was also lower than expected according to our previous studies (10.4% vs 18%; p = 0.002) (NMRL2002, unpublished data) even lower compared to recent European carriage studies with rates ranging from 23.2% - 34.2% for first- and second year students respectively, living in university residence halls in UK [19] and 22.5% among technical secondary school students 18 years of age in Italy [20]. This might be due to the fact that the majority of the university students in Greece rarely live in student residencies during their academic studies.

This lower carriage rate observed in both groups compared to our previous studies 20 years ago for recruits [12] and students (NMRL 2002, unpublished data) may be due to several factors such as reduction to smoking habits and vaccination.

A significant reduction on smoking habits was observed between the participants in previous and the current meningococcal carriage studies; 64.9% of the military recruits were smokers in the previous carriage study [12], while 55.7% reported to be active smokers in 2014 (p = 0.00016). Regarding the university students, a higher reduction on active smoking was observed; 47% vs 15.4% for the studies 2002 and 2015 respectively (p = 0.00).

MenC vaccination coverage, which found to be as high as 83.4% in adolescents in 2009 [21] and the introduction of MCV4 since 2011 could have contributed to the lower carriage rates in the present study. In addition to the well-known 67% reduction in the prevalence of serogroup C carriage one year after the introduction of MenC vaccine [8] recent evidence was provided by a clinical study regarding the impact of MCV4 vaccine on carriage among UK university students. According to this study, a 36.2% carriage reduction was observed for serogroups CWY while, a 39% reduction in carriage was observed for serogroup Y, 3–12 months after vaccination. [22].

MenB was predominant in both studied groups however, a remarkable two fold higher rate was observed among the military recruits, compared to our previous studies (44.7% vs 23%, p<0.05) while no change was observed among university students. This observation is of no surprise since during 1995–1999, MenB and MenC accounted for 43% (185/428) and 44% (187/428) of IMD cases respectively. Since 2000, MenB IMD cases increased dramatically from 49.6 (80/161) to 86.8% (46/53) (2012) with a simultaneous decrease of MenC cases from 12.4% (20/161) (2000) to zero (2012). In addition during 2013–2015 MenB IMD cases predominates accounting for 86.8%; 46/53 (2013), 66.7%; 38/57 (2014) and 79.6%; 39/49 (2015) while, only 9 MenC cases were identified during the above three years [16].

A significant increase among the MenY strains was observed among the university students, reaching 18.2% compared to previous studies in which no MenY was identified (NMRL 2002, unpublished data). The later, is in agreement with recent studies in UK showing a significant increase in MenY carriage among students ranging from 1.7% to 2.9% (1997 and 2009 respectively) [19], reaching 5.5% in the first year university students aged 19–25 in 2011 [23]. In addition, this genogroup was the second most frequent in the military recruits; a significant increase being observed in comparison to our previous studies (8.7% vs 2.8%, *p* = 0.015). It is worth noting, that until recently, MenY was of minor importance among the invasive strains in Europe accounting for less than 2% of reported IMD cases mainly isolated among the elderly. However, an increase of MenY cases has been reported in various European countries since 2010 and a shift to younger age groups was also observed [24]. Similarly, an increase was observed in Greece from 2% (2013) and 3.5% (2014) to 8.1% for 2015 [16]. The increase among carriers 18 to 19 years old underlines the potential of this genogroup to become important also in Greece.

An increase in MenW isolates among the recruits was observed in comparison to previous studies (2.9% vs 0.8%) and a decrease (6.5% vs 10.8%) among the university students; however, no statistical significance (p = 0.13 and p = 0.42 respectively) was observed.

As for MenC, only two of 180 isolates belonged to this genogroup (1.1%), both recovered from recruits, while none of the carrier isolates in the university student’s group belonged to this genogroup. This might be due to the introduction of MenC vaccine in Greece since 2001 and its effect on carriage, as according to their vaccination status response, 63.8% had been vaccinated (76.2% of univeristy students and 55% of recruits). Of note, the fact that the two MenC carriers reported to be vaccinated might indicate the phenomenon of waning immunity [25].

MenA, globally a leading cause of epidemic IMD, was not found in the present study. This is in line with the fact that MenA for a long time have only sporadically caused invasive disease in Europe and are rare in healthy individuals, although this particular genogroup was the most prevalent among the carrier isolates from ethnic Greek school children (1995) as well as from invasive isolates between 1999–2003 in Greece [26, 27].

Another interesting finding is the decrease in the proportion of the negative to genogroup specific PCR isolates compared to previous studies (42.2% vs 60%), with a further reduction (29.4%) due to the identification of cnl isolates by the application of the whole genome sequence analysis, highlighting its potential usefulness on the characterization of meningococcal carriage isolates.

Among the negative to genogroup spesific PCR isolates, MenE, a rather rare genogroup, was identified as the third most frequently isolated genogroup (8.3%) in both studied groups (7.8% among recruits and 9.1% among university students). To our knowledge, there is a limited number of published reports on its isolation from carriers; 7.5% of the isolates were identified as MenE in a recent carriage study among students 11–19 years old from Brazil [28].

Furthermore, 6 MenX isolates were identified. Although until 2013 there were no MenX IMD cases in Greece, one fatal case of IMD caused by this capsular group was reported in 2014 [16].

A known association between certain genogroups and particular clonal complexes was confirmed. The application of the MLST technique showed that the major types (sequence types, ST) prevailing in our country during the period 2004–2014 among the MenB IMD cases were the ST-269 (14,7%), ST-32 (10,5%), ST- 162 (5,7%) and to lesser extent 41/44cc (5.6%) [29]. The latter clonal complex, one of the most prevalent hyperinvasive lineage being associated mainly with the MenB IMD in Europe and elsewhere [30] was the most prevalent (30%) among the MenB asymptomatic carriers. This observation is of a great concern and must be under close surveillance since this particular lineage can potentially cause epidemic waves such as the New Zealand epidemic [31].

The clonal composition of the 53 cnl isolates was highly diverse represented mainly by 1136cc and 198cc. This comes in agreement with previous studies at a European level as well as studies in Latin America in which this diversity seem to be a stable feature [30, 32].

The inadequacy of data on MCV4 vaccination status, was one possible limitation in our study emphasizing the need for the introduction of a centralized immunization information system (enabling recording and recall) and the implementation of school entry mandates, as had been suggested previously [21].

Some other possible limitations could be that the sampling was performed in different season and years; military recruits (January 2014) and university students (May 2015). However, as shown previously, carriage rates do not appear to vary by season [33]. In addition, similar annual IMD incidence was notified for both years (0.61 and 0.59 / 100 000 for 2013 and 2014 respectively) [16]. Another possible limitation could be that sampling in the military group was performed in two stages (1^st^ and 7^th^ day of recruitment). However our results showed that there was no significant change on the carriage rate (13.1% vs 17.1% in both days respectively), (p = 0.14).

Despite these possible limitations, the present study provides an overview on the circulation of meningococcal strains isolated from individuals sharing a defined age group as well as a comparison to previous studies on the same groups 20 or more years earlier. Information provided on meningococcal carriage in Greek healthy young university students and military recruits, suggesting an overall decrease in meningococcal carriage rate.

Moreover, our findings, during the pre-MenB vaccination era in Greece, demonstrate a predominance of MenB among carriers with the prevalence of the hyperinvasive lineage 41/44 cc which was found to present a majority of carried meningococci

The increasing prevalence of MenB carriage among both university students and military recruits indicates the need of further investigations on the possible coverage of 4CMenB vaccine on these strains by Meningococcal Antigen Typing System (MATS) analysis as was carried out previously for invasive isolates [34]. Measurement of the expression of vaccine antigens in MenB carriage isolates in correlation with bactericidal titres might provide evidence of local immune response in the nasopharynx [35].

These data, could serve as basis for future comparisons and estimations as long term carriage studies are warranted for early detection of changing the genogroup distribution in countries where immunisation campaigns are either introduced or to be introduced. A future study possibly performed after the introduction of the 4CMenB vaccine in similar groups will be of great help for monitoring the impact of the aforementioned vaccine on nasopharyngeal carriage.

## Supporting Information

S1 Fig Map of the different geographical regions in Greece (DOCX)

S1 Table Reported vaccination status among the participants (DOCX)

S2 Table Participants by place of origin (DOCX)

S3 Table Univariate analysis for association between place of origin and meningococcal carriage (DOCX)
