# Supplementary figures and images for: Meningococcal Carriage in Military Recruits and University Students during the Pre MenB Vaccination Era in Greece (2014-2015)
Source: PLoS One. 2016 Dec 1;11(12):e0167404. doi: 10.1371/journal.pone.0167404 (PMC5131982; doi:10.1371/journal.pone.0167404)

**˝S1 Fig˝ Map of the different geographical regions in Greece**


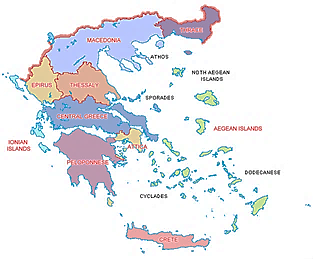

Supplement: S1 Fig — (DOCX) [file pone.0167404.s001.docx]
