# Supplementary material for: Meningococcal Carriage in Military Recruits and University Students during the Pre MenB Vaccination Era in Greece (2014-2015)
Source: PLoS One. 2016 Dec 1;11(12):e0167404. doi: 10.1371/journal.pone.0167404 (PMC5131982; doi:10.1371/journal.pone.0167404)
Supplement: S1 Table — (DOCX) [file pone.0167404.s002.docx]

**˝S1 Table˝ Reported vaccination status among the participants**

|  | **Reported vaccination status among the participants** | | | | | |
| --- | --- | --- | --- | --- | --- | --- |
|  | **Military recruits (n=680)** | | | **University students (n=740)** | | |
| **Vaccine** | **Vaccinated (%)** | **Unvaccinated (%)** | **UKN (%)** | **Vaccinated(%)** | **Unvaccinated(%)** | **UKN(%)** |
| **MenC** | 372 (54.7) | 25 (3.7) | 283 (41.6) | 534 (72.2) | 27(3.6) | 179(24.2) |
| **MCV-4** |  |  | 680 (100) | 63 (8.5) | 86 (11.6) | 591 (79.9) |
