# Supplementary material for: Meningococcal Carriage in Military Recruits and University Students during the Pre MenB Vaccination Era in Greece (2014-2015)
Source: PLoS One. 2016 Dec 1;11(12):e0167404. doi: 10.1371/journal.pone.0167404 (PMC5131982; doi:10.1371/journal.pone.0167404)
Supplement: S2 Table — (DOCX) [file pone.0167404.s003.docx]

**˝S2 Table˝** **Participants by place of origin**

| **Place of origin** | **University students** | **%** | **Recruits** | **%** | **total** | **%** |
| --- | --- | --- | --- | --- | --- | --- |
| Thrace (A) | 42 | **5.7** | 11 | **1.6** | 53 | **3.7** |
| Macedonia (B) | 124 | **16.7** | 69 | **10.1** | 193 | **13.6** |
| Thessaly (C) | 40 | **5.4** | 27 | **4.0** | 67 | **4.7** |
| Epirus (D) | 58 | **7.8** | 9 | **1.3** | 67 | **4.7** |
| Central Greece (E) | 20 | **2.7** | 23 | **3.4** | 43 | **3.0** |
| Attica (F) | 262 | **35.4** | 329 | **48.4** | 591 | **41.6** |
| Peloponnese (G) | 74 | **10.0** | 82 | **12.1** | 156 | **10.9** |
| Ionian Islands (H) | 8 | **1.1** | 10 | **1.5** | 18 | **1.3** |
| Aegean Islands (I) | 13 | **1.7** | 72 | **10.6** | 85 | **6.0** |
| Crete (J) | 78 | **10.5** | 33 | **4.9** | 111 | **7.8** |
| Abroad | 15 | **2.0** | 5 | **0.7** | 20 | **1.4** |
| Uknown | 6 | **0.8** | 10 | **1.5** | 16 | **1.1** |
| **Total** | **740** | **100** | **680** | **100** | **1420** | **100** |
