# Supplementary material for: Meningococcal Carriage in Military Recruits and University Students during the Pre MenB Vaccination Era in Greece (2014-2015)
Source: PLoS One. 2016 Dec 1;11(12):e0167404. doi: 10.1371/journal.pone.0167404 (PMC5131982; doi:10.1371/journal.pone.0167404)
Supplement: S3 Table — (DOCX) [file pone.0167404.s004.docx]

**˝S3 Table˝ Univariate analysis for association between place of origin and meningococcal carriage**

| **Place of Origin** | **Meningococcal Carrier** | | |
| --- | --- | --- | --- |
|  | **No** | **Yes** | **Total** |
| **Thrace (A)** | 48 | 5 | 53 |
| **Abroad** | 16 | 4 | 20 |
| **Macedonia(B)** | 163 | 30 | 193 |
| **Thessaly (C)** | 61 | 6 | 67 |
| **Epirus (D)** | 57 | 10 | 67 |
| **Central Greece (E)** | 36 | 7 | 43 |
| **Attica (F)** | 522 | 69 | 591 |
| **Peloponnese (G)** | 137 | 19 | 156 |
| **Ionian islands H)** | 14 | 4 | 18 |
| **Aegean islands (I)** | 71 | 14 | 85 |
| **Crete (J)** | 100 | 11 | 111 |
| **Not available** | 15 | 1 | 16 |
| **Total** | 1240 | 180 | 1420 |

| **Chi-Square Tests** | | | |
| --- | --- | --- | --- |
|  | Value | df | Asymp. Sig. (2-sided) |
| Pearson Chi-Square | 9,076^a^ | 11 | ,615 |
| Likelihood Ratio | 8,816 | 11 | ,639 |
| N of Valid Cases | 1420 |  |  |
| a. 3 cells (12,5%) have expected count less than 5. The minimum expected count is 2,03. | | | |
